# Endogenous Antioxidant and LOX-Mediated Systems Contribute to the Hepatoprotective Activity of Aqueous Partition of Methanol Extract of *Muntingia calabura* L. Leaves against Paracetamol Intoxication

**DOI:** 10.3389/fphar.2017.00982

**Published:** 2018-02-15

**Authors:** Zainul Amiruddin Zakaria, Nur Diyana Mahmood, Siti Syariah Mamat, Nurliana Nasir, Maizatul Hasyima Omar

**Affiliations:** ^1^Laboratory of Halal Science Research, Halal Products Research Institute, Universiti Putra Malaysia, Seri Kembangan, Malaysia; ^2^Department of Biomedical Sciences, Faculty of Medicine and Health Sciences, Universiti Putra Malaysia, Seri Kembangan, Malaysia; ^3^Phytochemistry Unit, Herbal Medicine Research Centre, Institute for Medical Research, Kuala Lumpur, Malaysia

**Keywords:** *Muntingia calabura* leaves, paracetamol-induced hepatotoxicity, hepatoprotective activity, endegenous antioxidant activity, anti-inflammatory activity

## Abstract

Methanol extract of *Muntingia calabura* L. (family Muntingiaceae) leaf has been reported to exert various pharmacological activities including hepatoprotection. The present study was carried out to identify the most effective hepatoprotective partition derived from the extract and to determine the mechanisms of action involved. The extract was partitioned using solvents with different polarity to yield petroleum ether (PEMC), ethyl acetate (EAMC), and aqueous (AQMC) extracts. Each extract, at 250 mg/kg, was subjected to the paracetamol (PCM)-induced hepatotoxic assay and several parameters such as liver weight, liver/body weight ratio, serum liver enzymes' level, and histopathological examinations were determined. Each partition was also tested for their antioxidant and anti-inflammatory potentials. The most effective extract (AQMC) was prepared in additional dose of 50 and 500 mg/kg, and then subjected to the same liver toxicity test in addition to the endogenous antioxidant enzymes assay. Moreover, AQMC was also subjected to the phytochemical screening and HPLC analysis. Overall, from the results obtained: AQMC exerted significant (*p* < 0.05): (i) antioxidant activity when assessed using the DPPH, SOD and ORAC assays with high TPC detected; (ii) anti-inflammatory activity via LOX, but not XO pathway; (iii) hepatoprotective activity indicated by its ability to reverse the effect of PCM on the liver weight and liver/body weight ratio, the level of serum liver enzymes (ALT, AST, and ALP), and activity of several endogenous antioxidant enzymes (SOD and CAT). Phytochemicals analyses demonstrated the presence of several flavonoid-based bioactive compounds such as gallic acid and quercetin, which were reported to possess hepatoprotective activity. In conclusion, AQMC exerts hepatoprotective activity against the PCM-induced toxicity possibly by having a remarkable antioxidant potential and ability to activate the endogenous antioxidant system possibly via the synergistic action of its phytoconstituents.

## Introduction

Drug-induced hepatotoxicity (DIH) is an impending problem which involve a vast number of drugs and chemical compounds including prescribed medications, as liver is a fundamental organ in metabolic disposition of virtually all drugs and foreign substances (Suk and Kim, 2012). As these compounds are metabolized in liver microsomes, their excessive consumption can trigger liver damage that result in abnormalities of the liver function. Paracetamol (PCM)-induced liver injury (PILI) is a regular example of DIH that still remains as a global issue, especially in the United Kingdom (UK), Europe, United States (US), and Australasia. PCM overdose is known to cause a range of hepatic damages from mild to severe hepatotoxicity, leading to acute liver failure (ALF) and death, either accidentally or unintentionally due its wide availability and accessibility (Marzilawati et al., 2012). Nevertheless, the incidence of PILI in Malaysia was reported to be at low rates (<10%) (Mohd Zain et al., 2006; Marzilawati et al., 2012).

Current treatment options for common liver injury are less effective. Although there have been remarkable progress in discovering treatment for liver injury over the last several decades, most of the therapies still did not yield satisfactory outcomes in patients (Dhiman et al., 2012). The effectiveness of currently available medications, such as colchicine, corticosteroids and interferon, are conflicting at best and the occurrence of adverse effects are profound (Ghosh et al., 2011). All too often the treatment is worse than the disease. Due to these issues, which was worsen by the scarce treatment options, novel prophylactic and effective therapeutic agent with low incidence of adverse effects are urgently needed. Interestingly, herbals and medicinal plants exert the potential to constitute such a group (Hong et al., 2015). A wide range of herbals and medicinal plants have been utilized either in the form of extract of single plant or compound preparations of more than one-plant types to treat liver injury (Rajaratnam et al., 2014).

One of the medicinal plants that have been reported to ameliorate liver injury is *Muntingia calabura* L., a tropical plant species belong to the family of Muntingiaceae. Although there is a lack of report on the medicinal uses of this plant in Malay traditional culture, *M. calabura* has been traditionally used by the Peruvians, for example, to treat gastric ulcer, prostate gland swelling, headache and cold. Scientific studies have confirmed that the plant possess a wide range of pharmacologic activities (Mahmood et al., 2014b), including remarkable antioxidant (Zakaria, 2007; Zakaria et al., 2011) and anti-inflammatory activities (Zakaria et al., 2007). Recent studies have revealed that the antiulcer effect of *M. calabura* extract could be attributed partly to the plant's high antioxidant capacity and marked anti-inflammatory activity (Zakaria et al., 2014; Balan et al., 2015) and involve, in part, the enhancement of endogenous antioxidant system (Balan et al., 2015; Zakaria et al., 2016). Taking into account the strong association between high antioxidant and anti-inflammatory activities with potent hepatoprotective effect (Chattopadhyay, 2003), the ability of *M. calabura* to impede liver injury was investigated. In our previous study, the methanol extract of *M. calabura* (MEMC) leaves had been reported to ameliorate PILI in rats and this activity was associated with its remarkable antioxidant capacity and the presence of rutin, fisetin and quercetin (Mahmood et al., 2014a). To further study on the plant hepatoprotective activity, the MEMC was further extracted using solvents of different polarities via a process known as partition that aimed to separate the bioactive compounds presence in MEMC into non-polar, intermediate and polar compounds. The respective petroleum ether, ethyl acetate and distilled water were used for this purpose. Thus, the objectives of the present study were to identify extract with the most effective hepatoprotective activity against PILI in rats; to elucidate the possible hepatoprotective mechanisms involved; and to determine the possible bioactive compounds presence in the most effective partition.

## Methodology

### Extraction and fractionation of *M. calabura* leaves

Method described by Zakaria et al. (2011) and Sufian et al. (2013) was employed to prepare the crude extract of *M. calabura* leaves and its fractions, respectively. Matured leaves (500 g) were air-dried at room temperature (27 ± 72°C) for 1–2 weeks and ground into fine powder. The powder was soaked in methanol (MeOH) at a ratio of 1:20 (w/v) for 72 h. The mixture was filtered using cotton wools followed by Whatman No. 1 filter papers. The soaking and filtration processes were repeated for another two times using the collected residue. The filtrate collected from each extraction was pooled and evaporated in a rotary evaporator at 40°C under reduced pressure to obtain methanol extract of *M. calabura* (MEMC). The dried crude extract obtained was weighed and then suspended in MeOH before being added with distilled water (dH_2_O) to afford an aqueous MeOH solution. The solution was then sequentially partitioned with solvents of different polarity, namely petroleum ether (PE; non-polar) and ethyl acetate (EA; intermediate polar), to yield the respective petroleum ether fraction (PEF) and ethyl acetate fraction (EAF). The final solution, which did not dissolve in PE or EA and represents the polar fraction, was the aqueous fraction (AQF) of MEMC and was also collected. All collected fractions were filtered and then PEF and EAF were evaporated to dryness at 40°C under reduced pressure while AQF was kept at −80°C for at least 48 h before being subjected to the freeze-drying process. Finally, all dried fractions were dissolved in 10% DMSO to prepare the final concentration for each extract.

### Antioxidant profiling of PEMC, EAMC, and AQMC

#### Total phenolic content

Determination of total phenolic content (TPC) was performed using Folin-Ciocalteu reagent according to the method of Singleton and Rossi (1965) with slight modifications. The respective fraction (1 mg) was extracted on a shaker at 200 rpm for 2 h with 1.0 ml 80% methanol containing 1.0% hydrochloric acid and 1.0% dH_2_O at room temperature. The mixture was then centrifuged at 6,000 rpm for 15 min and the supernatant obtained was decanted into vials. The supernatant of each fraction (200 μl) was mixed with 400 μl Folin-Ciocalteu reagent (0.1 ml/0.9 ml) and allowed to stand at room temperature for 5 min. Then, 400 μl sodium bicarbonate (60.0 mg/ml) solution was added, and the mixture were allowed to stand at room temperature for 90 min. Absorbance was measured at 725 nm. A calibration curve was generated by using the optical density (OD) of gallic acid (GA) standard and the level in the samples were expressed as GA equivalents (GAE)-TPC mg/100 g.

#### Diphenylpicrylhydrazyl (DPPH) radical scavenging assay

The DPPH radicals scavenging assay was estimated according to the method of Blois (1958) with slight modifications. Briefly, the sample was serially diluted to obtain final concentrations of 3.12, 6.25, 12.5, 25, 50, 100, and 200 μg/ml solutions from 1.0 mg/ml stock sample. Approximately 50 μl of the previously prepared solutions was added to 50 μl of DPPH (FG: 384.32) (1 mM in ethanolic solution) and 150 μl of absolute ethanol in a 96-well microtiter plate in triplicate. The plate was shaken (15 s; 500 rpm) and left to stand at room temperature for 30 min. The absorbance of the resulting solution was measured spectophotometrically at 520 nm. Green tea, in different concentrations (3.13–200 μg/ml), was used as a standard. The lower absorbance of reaction mixture indicates the higher free radical scavenging activity. The results were expressed as percentage inhibition (*I* %) using the following equation:

$$I\%=({\text{Abs}}_{\text{control}}-{\text{Abs}}_{\text{sample}}/{\text{Abs}}_{\text{control}})\times 100$$

Where, Abs_control_ is the absorbance of the control and Abs_sample_ is the absorbance of the sample. The IC_50_ was obtained by linear regression analysis of dose-response curve plotting between *I*% and concentrations.

#### Superoxide anion scavenging assay

A superoxide anion (SOA) radical scavenging assay was carried out to measure the xanthine oxidase (XO)-inhibiting activity of PEMC, EAMC, and AQMC, and was performed according to the method of Chang et al. (1996) with slight modifications. Basically, nitroblue tetrazolium (NBT) solution (100 ml of 4.1 mM/l) was prepared by adding 3.15 g Tris-HCl, 0.1 g MgCl_2_, 15.0 mg 5-bromo-4-chloro-3-indolyl phosphate, and 34.0 mg 4-NBT chloride to 100 ml distilled water. The reaction mixture (100 ml) was prepared by dissolving 0.53 g NA_2_CO_3_ (pH 10.2), 4.0 mg EDTA, and 50.0 mg xanthine in 0.025 mM NBT solution. The mixture was kept refrigerated at 4°C. The negative control contained reaction mixture (999 ml), which was transferred into a microcuvette and placed in a 25°C cell holder in a spectrophotometer to auto zero. Superoxides were generated by adding 1.0 ml XOD (20 U/ml). After mixing by manual shaking thoroughly for 5 s, OD measurement were taken at 560 nm for 120 s using a spectrophotometer (Lambda 2S). Sample solutions were added to 799 ml reaction mixture and measured as above.

#### Oxygen radical absorbance capacity (ORAC) test

The oxygen radical absorbance capacity (ORAC) test was carried out according to Huang et al. (2002) with slight modifications. Briefly, 175 μl of the sample/blank were dissolved with Phosphate Buffer Solution (PBS; pH 7.4) at the concentration of 160 μg/ml. To 96-well microplates, 25 μl each of samples or blank (solvent/PBS) were added. Then, 150 μl of 1 mM fluorescent sodium salt solution was added and the plate was incubated for 10 min at 37°C. Approximately 25 μl of 240 mM 2, 20-azobis (2-amidinopropane) dihydrochloride (AAPH) solution was added to make up a total volume of 200 μl/well. The solution was mixed by shaking at maximum intensity for 50 s. Fluorescence was monitor kinetically, recorded every 60 s at 37°C until it reached 0 (excitation at 485 nm, emission at 528 nm) using a fluorescence spectrophotometer (BMG FLUOstar Omega, UK) equipped with an automatic thermostatic autocell-holder. Data were collected every 1 min for 2 h and were analyzed using MARS Data Analysis Reduction 2.0 Software (BMG LABTECH, Germany). On each plate, different dilutions of Trolox were used as reference standard. ORAC values were expressed as mmol of Trolox Equivalents (TEs) per gram of dry weight.

### *In vitro* anti-inflammatory profiling of PEMC, EAMC, and AQMC

#### Xanthine oxidase (XO) assay

The spectrophotometric method was used to measure the xantine oxidase (XO) inhibiting activity of the fractions as described by Noro et al. (1983). A mixture of 130 μl potassium phosphate buffer (0.05 M, pH 7.5), 10 μl sampels and 10 μl XO solution were incubated at 25°C for 10 min. The addition of 100 μl substrate (xanthine solution) initiates the enzymatic reaction that promotes the conversion of xanthine to uric acid and hydrogen peroxides. This reaction was measured at the absorbance of 295 nm. The samples and reference standards were dissolved in 10% DMSO. All reactions were performed in triplicates in a 96-well microplate.

#### Lipoxygenase (LOX) assay

Lipoxygenase (LOX) inhibiting activity was measured using the spectrophotometric method as described by Azhar-Ul-Haq et al. (2004). A mixture of 160 ml sodium phosphate buffer (0.1 M, pH 8.0), 10 ml samples and 20 ml of soybean LOX solution were incubated for 10 min at 25°C. The reaction was initiated with an addition of 10.0 ml substrate (sodium linoleic acid solution) resulting in the enzymatic conversion of linoleic acid to form (9Z, 11E)-(13S)-13-hydroperoxyoctadeca-9, 11-dienoate, which was measured at 234 nm over a period of 6 min. All samples, namely PEMC, EAMC, AQMC, and NDGA (reference standards), were dissolved in methanol. All reactions were performed in triplicates in the 96-well microplate.

#### Animals

Healthy male Sprague Dawley rats (8–9 weeks old; weighing 180–220 g) were used throughout the study. Animal were obtained from the Animal House Facility, Faculty of Medicine and Health Sciences, Universiti Putra Malaysia. They were housed at room temperature (27 ± 2°C) and allowed free access to food and tap water *ad libitum*. The animals were acclimatized to laboratory conditions for 7 days before the commencement of experiments. This study was carried out in accordance with the recommendations of the current UPM guidelines for the care of laboratory animals and the ethical guidelines for investigations of experimental pain in conscious animals. The protocol was approved by the International Animal Care and Use Committee (IACUC), Faculty of Medicine and Health Sciences, UPM (Ethical approval no.: UPM/FPSK/PADS/BR-UUH/00449). All experiments were conducted between 09.30 and 18.30 h to minimize the effects of environmental changes.

#### Paracetamol-induced hepatotoxic assay

The *in vivo* hepatoprotective activity of MEMC was determined using the PCM-induced hepatotoxic assay in rats. The animals (*n* = 6) were randomly divided into 8 experimental groups and administered with the test solutions as described below:

- Group I served as the normal control—received 10% DMSO.
- Group II served as the negative control—received 10% DMSO.
- Group III served as the positive control—received 50 mg/kg N-acetyl cysteine (NAC).
- Pre-treatment groups received different type of partition at the dose of 250 mg/kg as described below:
  - Group IV received 250 mg/kg PEMC,
  - Group V received 250 mg/kg EAMC and,
  - Group VI received 250 mg/kg AQMC.
- To establish the hepatoprotective profile of the most effective partition (determined later to be AQMC), two additional groups were treated with AQMC at the doses of 50 and 500 mg/kg as described below:
  - Group VII received 50 mg/kg AQMC and,
  - Group VIII received 500 mg/kg AQMC.

The animals were fasted for 48 h prior to the experiment under standard laboratory condition but allowed accessed to distilled water (dH_2_O) *ad libitum*. After 48 h, each group of rats received the respective dose of test solution orally once daily for 7 consecutive days. The oral administration of PCM was performed 3 h after the last DMSO/NAC/partitions administration on the 7th day except for group I, which received only 10% DMSO. Forty eight (48) h after the hepatic injury induction, the animals were anesthetized using diethyl ether and the blood was drained for biochemical parameters study. The animals were then sacrificed by cervical dislocation and the liver was removed for histopathological examinations.

#### Biochemical analysis

Biochemical parameters were assayed according to the standard methods. The enzymes alanine transferase (ALT), aspartate aminotransferase (AST), and alkaline phosphatase (ALP) were measured using a Hitachi 902 Automatic Chemical Analyser (Japan).

#### Histophatology

After the liver tissue was fixed in 10% formalin, specimens were embedded in paraffin, sectioned (3–5 μm), and stained with hematoxylin and eosin. The histochemical sections were evaluated under a light microscope by a pathologist and the severity of hepatic injury was scored according to the method described by El-Beshbishy et al. (2010).

### Assessment of the liver's endogenous antioxidant enzymes activity

#### Preparation of liver homogenates

Liver homogenates were prepared by mincing and homogenizing 100 mg of liver tissue in 1 ml cold PBS buffer using a steel homogenizer. The homogenate was collected and then centrifuged using Sorvall™ Legend™ Micro 17R microentrifuge (Thermo Fisher Scientific) at 4,000 rpm and 4°C for 25 min. The resultant supernatant was used for the determination of antioxidant enzymes' activity.

#### Superoxide dismutase (SOD) activity

Superoxide dismutase activity of the respective supernatant was measured using the Cayman's Assay Kit (Cayman Chemical, USA) according to the protocol provided by the manufacturer. Basically, the SOD standard was prepared in several concentrations (0, 0.005, 0.01, 0.02, 0.03, 0.04, and 0.05 U/ml). Approximately 10 μl of each standard or sample was added into each well followed by 200 μl of diluted radical detector. The reactions were initiated by adding 20 μl of diluted xanthine oxidase to all the wells that were used. The plate was then covered and incubated on a shaker for 30 min at room temperature. Absorbance was measured at 440–460 nm using ELISA reader (Asys UVM 340, UK). Unit of the SOD activity were defined as the amount of enzyme needed to exhibit 50% dismutation of the superoxide radical and the SOD activity was expressed as units per gram tissue.

#### Catalase (CAT) activity

Catalase activity of the respective supernatant was measured using the Cayman's Assay Kit (Cayman Chemical, USA) according to the protocol provided by the manufacturer. Basically, the formaldehyde standard were prepared in several concentrations (0, 5, 15, 30, 45, 60, and 75 μM). Approximately 20 μl of each formaldehyde standard or samples was added into each well followed by 30 μl of methanol and 100 μl of diluted assay buffer. Then, about 20 μl of diluted hydrogen peroxide were added to all used wells to initiate the reactions. The plate was later covered and incubated on a shaker for 20 min at room temperature. After that, 30 μl of diluted potassium hydroxide were added to each well to terminate the reaction and then 30 μl of CAT purpald (chromogen) was added to each well. The plate was incubated on a shaker for 10 min at room temperature. Lastly, 10 μl of catalase potassium periodate was added to each well and the plate was incubated on a shaker for 5 min at room temperature. Absorbance was measured at 540 nm using ELISA reader (Asys UVM 340, UK). Units of CAT were expressed as the amount of enzyme that caused the formation of 1.0 nmol of formaldehyde per min at 25°C and the CAT activity was expressed in terms of unit per gram tissue.

#### Chemicals and reagents for HPLC and ultra-HPLC-electrospray ionization-mass spectrometry (UHPLC-ESI-MS) analyses of AQMC

Formic acid, methanol and LCMS grade acetonitrile were purchased from MERCK (Malaysia). HPLC grade water was prepared from distilled water using a Milli-Q-system (Millipore, MA) and was used during analytical UHPLC analysis. Gallic acid, kaempferol, kaempferol-3-*O*-glucoside, and quercetin were purchased from Sigma (USA). All of other solvents and chemicals used in this study were of analytical grade. Stock and working standards were prepared by dissolving these analytes in 100% methanol. The standard solutions were stored at 4°C and are stable for at least 3 months.

#### HPLC profiling of AQMC and comparison of peaks with several standard flavonoids

HPLC analyses were performed with Dionex Ultimate 3000 liquid chromatograph (Thermo Scientific, USA) equipped with vacuum degasser, an–autosampler, a binary pump, and a UV-vis detector. The separation was performed on a Kinetex (Phenomenex, USA) C18 analytical column (4.6 × 150 mm, 5 μm particle size) at flow rate 1 ml/min. The temperature of the column was maintained at 40°C and the injection volume was 10 μl. Samples were filtered by 0.2 μm PTFE filter prior to injection. The mobile phases used were water with 0.1% Formic acid; mobile phase A and methanol with 0.1% Formic acid; mobile phase B. The separation was conducted using the following multi-gradient step: 0 min 5% B; 15 min, 15% B; 15 min 45% B; 5 min 90%B; and finally a conditioning cycle of 5 min with the initial conditions for the next analysis. The UV-vis detection was performed in a wavelength range 200–600 nm.

#### UHPLC-ESI-MS profiling of AQMC

The UHPLC system was performed on a Dionex 3000 UHPLC system acquired from Thermo Fisher Scientific (USA) that consisted of an autosampler equipped with a column oven, a tray compartment cooler, and a binary pump with built in solvent degasser. The chromatographic separation was performed on a BEH C18 UHPLC column, 100 mm × 2.5 μm, 1.7 μm (WATERS) at a flow rate of 0.3 ml/min. The mobile phases used were (A) 0.1% formic acid in water and (B) 0.1% formic acid in acetonitrile. The gradient started with 5% mobile phase B at 5 min, reaching 15% mobile phase B at 20 min, 40% mobile phase B at 50.0 min, at isocratic elution of 90% B for 3 min. The gradient reached the initial conditions were held for 2 min as a re-equilibration step. The injection volume was 10 μl and the column temperature was maintained at 40°C.

The UHPLC system was coupled to a linear ion-trap-Orbitrap mass spectrometer Q Exactive from Thermo Fisher Scientific (U.S.A) equipped with an electrospray ionization (ESI) source. The mass detection was performed in a range of 150–1,500 m/z. The ESI source was operated in negative ion mode under the following specific conditions: source voltage, 3.2 kV; sheath gas, 35 arbitrary units; auxiliary gas, 15 arbitrary unit; sweep gas, 10 arbitrary unit; and capillary temperature, 320°C. Nitrogen (>99.98%) was employed as sheath gas, auxiliary and sweep gas. Instrument control and data acquisition were performed with Chameleon 6.8 software and Xcalibur 2.2 software (Thermo Fisher Scientific).

### Statistical analysis

All data were presented as mean ± SEM. Statistical analysis was performed using GraphPad Prism version 5. Data obtained were analyzed using the one-way analysis of variance (ANOVA) and, the differences between groups and the control group was determined using Dunnett's *post hoc* test with *P* < 0.05 as the limit of significance.

## Result

### Comparison of antioxidant, anti-inflammatory and hepatoprotective potentials of PEMC, EAMC, or AQMC at the dose of 250 m/kg

#### *In vitro* antioxidant and anti-inflammatory activities of PEMC, EAMC, or AQMC

The antioxidant and anti-inflammatory intensities of PEMC, EAMC, and AQMC are shown in Table 1. From the results obtained, (i) EAMC possessed the highest TPC value, which is an approximately 2-fold increase of the value obtained from PEMC and AQMC; (ii) EAMC and AQMC demonstrated the lowest IC_50_ value (<30 mg/100 g GAE) in comparison to AQMC (<70 mg/100 g GAE) when assessed for their ability to scavenge the DPPH activity; (iii) all partitions demonstrated remarkable percentage of scavenging activity against the SOA activity; (iv) EAMC demonstrated the highest ORAC value followed by AQMC and PEMC; (v) EAMC showed the highest percentage inhibitory activity against the XO assay, and; (vi) PEMC and EAMC demonstrated the highest percentage of inhibitory activity against the LOX assay.

**Table 1:** Antioxidant and anti-inflammatory activities of various partitions derived from methanol extract of *M. calabura* (MEMC), namely petroleum ether- (PEMC), ethyl acetate- (EAMC), or aqueous- (AQMC) partition of *M. calabura*.

	**ANTIOXIDANT**				**ANTI-INFLAMMATORY**	
	**TPC (mg/100 g GAE)**	**IC50 DPPH scavenging (μg/Ml)**	**SOD scavenging (%)**	**ORAC value (TE/100 g)**	**XO inhibition (%)**	**LOX inhibition (%)**
Green tea		13.90 ± 0.47				
NDGA						99.86 ± 0.14
Allopurinol					97.58 ± 0.32	
PEMC	447.09 ± 11.99	69.01 ± 0.74	99.40 ± 0.6	97,000	8.76 ± 2.80	100.0 ± 0.00
EAMC	871.71 ± 8.27	28.32 ± 0.74	94.67 ± 1.07	130,000	72.81 ± 2.52	95.54 ± 4.46
AQMC	413.56 ± 7.95	27.39 ± 0.74	100.00 ± 0.00	105,000	21.54 ± 4.95	84.40 ± 7.85

*TPC, Total phenolic content; DPPH, Diphenylpicrylhydrazyl; SOA, Superoxide anion; ORAC, Oxygen radical absorbance capacity; XO, Xanthine oxidase; LOX, Lipoxygenase; NDGA, Nordihydroguaiaretic acid*.

#### Effect of PEMC, EAMC, or AQMC on body and liver weights of rats intoxicated with PCM

Table 2 shows the effect of PEMC, EAMC, and AQMC on the body and liver weights, and liver/body weight ratio of rats following the oral administration of PCM. Oral administration of PCM (Group 2) caused significant (*p* < 0.05) increase in the liver, but not body, weight of the rats when compared to the normal (treated with 10% DMSO) group (Group 1). Pretreatment of rats with 250 mg.kg AQMC, but not PEMC or EAMC, significantly (*p* < 0.05) reversed PCM effects on the liver, thus, reduced the weight of liver and decreased the ratio of liver/body weight.

**Table 2:** Effect of PEMC, EAMC, or AQMC, at the dose of 250 mg/kg, on the body and liver weights, and liver/body weight ratio following the oral administration of paracetamol (PCM) in rats.

**Treatment**	**Dose (mg/kg)**	**Body weight (BW) (g)**	**Liver weight (LW) (g)**	**LW/BW (%)**
10% DMSO + 10% DMSO	–	207.90 ± 4.74	6.19 ± 0.36	2.97 ± 0.11
10% DMSO + PCM		205.50 ± 8.26	8.80 ± 0.73^a^	4.28 ± 0.24^a^
NAC + PCM	50	203.60 ± 2.70	7.05 ± 0.40^a^^b^	3.46 ± 0.15^a^
PEMC + PCM	250	197.70 ± 5.92	7.48 ± 0.41^a^	3.92 ± 0.25^a^
EAMC + PCM	250	195.20 ± 2.61	8.01 ± 0.30^a^	4.10 ± 0.17^a^
AQMC + PCM	250	201.90 ± 3.77	5.94 ± 0.32^b^	2.94 ± 0.17^b^

*Values are expressed as means ± S.E.M. of six replicates*.

a *Significant different as compared to normal control, P < 0.05*.

b *Significant different as compared to negative control (10% DMSO + PCM), P < 0.05*.

*NAC, N-acetyl cysteine*.

#### Microscopic observation and histopathological scoring of PCM-intoxicated liver tissue pre-treated with PEMC, EAMC, or AQMC

**Figure 1:**
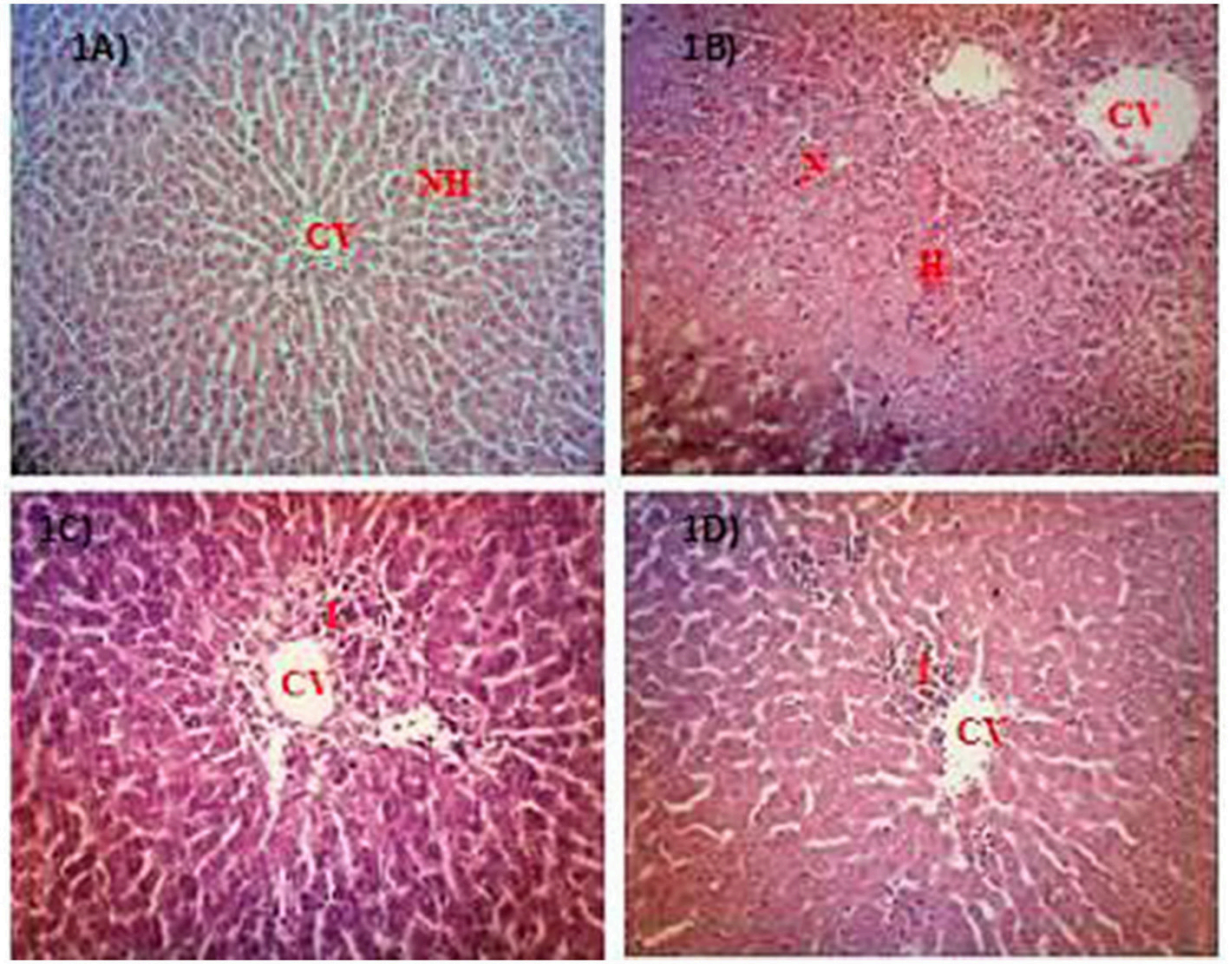
Microscopic examination of PCM-intoxicated rat's liver tissues pretreated orally with PEMC, EAMC or AQMC at the dose of 250 mg/kg. Photomicrographs show the liver tissue section of: **(A)** normal group (untreated), which showed normal liver architecture consisting of normal hepatocytes (NH) and central vein (CV); **(B)** hepatotoxic group (orally treated with only 3 g/kg PCM), which shows massive necrosis (N), hemorrhage (H) and inflammation (I); **(C)** positive group (orally pre-treated with 50 mg/kg NAC followed by PCM), which showed preservation of NH with mild I; **(D)** AQMC-pre-treated group followed by PCM, which showed normal liver architecture with mild I. PEMC and EAMC did not show hepatoprotective activity (data not shown). (100x magnification). NH, normal hepatocyte; CV, central vein; N, necrosis; I, inflammation; H, hemorrhage.

The microscopic examination and histopathological scoring of rat's liver tissue pre-treated with PEMC, EAMC or AQMC followed by the oral administration of PCM are shown in Figures 1A–D and Table 3. From the results obtained, AQMC, at 250 mg/kg, was found to cause remarkable attenuation of liver injury indicated by the absence of necrosis and hemorrhage when assessed at the microscopic level. The other partitions, PEMC and EAMC, failed to attenuate PILI indicated by the presence of severe necrosis and, moderate inflammation and hemorrhage.

**Table 3:** Histopathological scoring of PCM-intoxicated rat's liver tissue following pretreatment with PEMC, EAMC, or AQMC at the dose of 250 mg/kg.

**Treatment**	**Dose (mg/kg)**	**Steatosis**	**Necrosis**	**Inflammation**	**Hemorrhage**
Normal	−	–	–	–	–
10% DMSO + PCM		–	+++	++	++
NAC + PCM	50	–	+	+	–
PEMC + PCM	250		+++	++	++
EAMC + PCM	250		+	+	++
AQMC + PCM	250		–	+	–

*The severity of various features of hepatic injury was evaluated based on those following scoring scheme: − normal, + mild effect, ++ moderate effect, +++ severe effect*.

#### Effect of PEMC, EAMC, or AQMC on the level of serum liver enzymes of PCM-intoxicated rats

The effect of PEMC, EAMC, or AQMC on the level of serum liver enzymes, namely ALT, AST, and ALP, are shown in Table 4. The oral administration of PCM (Group 2) caused significant (*p* < 0.05) elevation in the level of ALT, AST, and ALP in comparison to the normal group (Group 1). Pre-treatment with 250 mg/kg AQMC caused remarkable (*p* < 0.05) decrease in the level of all enzymes while pre-treatment with PEMC or EAMC failed to affect any of the enzymes level. In addition, 50 mg/kg NAC also caused significant (*p* < 0.05) reduction in the level of ALT and AST, but not ALP, which is comparable to the activity of AQMC. Interestingly, the intensity of reduction of 250 mg/kg AQMC was higher than that of 50 mg/kg NAC.

**Table 4:** Effect of the orally administered PEMC, EAMC, or AQMC, at the dose of 250 mg/kg, on the level of serum liver biomarkers, namely alanine transferase (ALT), aspartate aminotransferase (AST), and alkaline phosphatase (ALP), of PCM-intoxicated rats.

**Treatment**	**Dose (mg/kg)**	**ALT (U/L)**	**AST (U/L)**	**ALP (U/L)**
10% DMSO + 10% DMSO	–	136.1 ± 10.52	124.39 ± 16.14	193.03 ± 41.44
10% DMSO + PCM		1714.0 ± 142.23^a^	2266.27 ± 340.41^a^	330.06 ± 42.35^a^
NAC + PCM	50	884.2 ± 195.47^a^^b^	1569.07 ± 106.47^a^^b^	284.31 ± 5.54
PEMC + PCM	250	1549.0 ± 336.51	2788.23 ± 199.33	371.08 ± 41.20
EAMC + PCM	250	1780.00 ± 111.52	2261.11 ± 422.37	331.55 ± 47.16
AQMC + PCM	250	297.7 ± 160.91^b^	431.28 ± 16.42^a^^b^	218.72 ± 21.35^b^

*Values are expressed as means ± S.E.M. of six replicates*.

a *Significant different as compared to normal control, P < 0.05*.

b *Significant different as compared to negative control (10% DMSO + PCM), P < 0.05*.

#### Selection of the most effective partition for further hepatoprotective study

From the data obtained when screening all partitions at 250 mg/kg, AQMC was found to show the most effective antioxidant and anti-inflammatory activities. In addition, AQMC also demonstrated the most effective hepatoprotective activity indicated by its ability to: (i) reduce the liver weight and liver/body weight ratio; (ii) show the lowest histopathological scoring supported by the microscopic liver tissue examination, and; (iii) remarkably reduced the level of serum liver enzymes, particularly ALT and AST. Thus, further examination was carried out on AQMC at 50 and 500 mg/kg to complete its hepatoprotective profiling.

#### Hepatoprotective profile of AQMC

**Figure 2:**
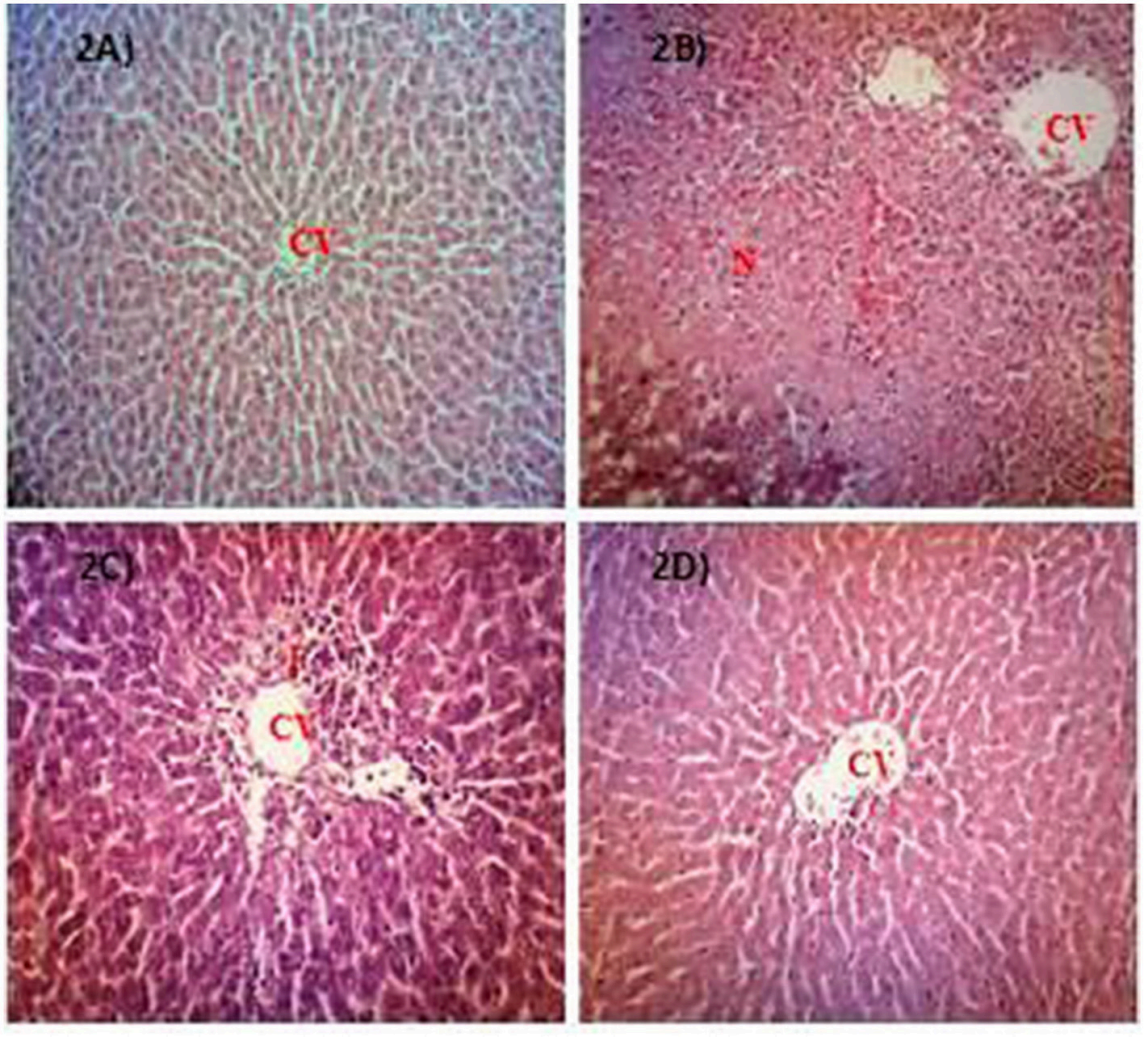
Microscopic examination of PCM-intoxicated rat's liver tissues pretreated orally with 50, 250, and 500 mg/kg AQMC. Photomicrographs show the liver tissue section of: **(A)** normal group (untreated), which showed normal liver architecture consisting of normal hepatocytes (NH) and central vein (CV); **(B)** hepatotoxic group (orally treated with only 3 g/kg PCM), which shows massive necrosis (N), hemorrhage (H) and inflammation (I); **(C)** positive group (orally pre-treated with 50 mg/kg NAC followed by PCM), which showed preservation of NH with mild I; **(D)** 500 mg/kg AQMC-pretreated group followed by PCM, which showed normal liver architecture with no significant changes observed. Groups pre-treated with 50 or 250 mg/kg AQMC followed by PCM showed only mild I (data not shown). 100x magnification, CV, central vein; N, necrosis; I, inflammation.

Table 5 shows the capability of AQMC to significantly (*p* < 0.05) reversed PILI in a dose-dependent evidenced by the partition ability to reduced the liver weight and liver/body weight ratio. Interestingly, AQMC did not affect the body weight throughout the experiment. Microscopic examinations and histopathological scoring of rat's liver tissue intoxicated with PCM following pretreatment with AQMC, in the dose range of 50–500 mg/kg, are shown in the respective Figure 2 and Table 6. From the results obtained, all doses of AQMC ameliorated PILI evidenced by the absence of necrotic and hemorrhagic tissues and the presence of inflamed tissues. These observations were further supported by the histopathological scoring that detected the absence of necrosis and hemorrhage in the liver tissue pre-treated with AQMC.

**Table 5:** Effect of AQMC, at the doses ranging between 50 and 500 mg/kg, on the body and liver weights, and liver/body weight ratio of PCM-intoxicated rats.

**Treatment**	**Dose (mg/kg)**	**Body weight, BW (g)**	**Liver weight, LW (g)**	**LW/BW (%)**
Normal	–	207.90 ± 4.74	6.19 ± 0.36	2.97 ± 0.11
10% DMSO + PCM		205.50 ± 8.26	8.80 ± 0.73^a^	4.28 ± 0.24^a^
NAC + PCM	50	203.60 ± 2.70	7.05 ± 0.40^a^^b^	3.46 ± 0.15^a^^b^
AQMC + PCM	50	206.30 ± 5.23	7.58 ± 0.19^b^	3.67 ± 0.17^a^^b^
	250	201.90 ± 3.77	5.94 ± 0.32^b^	2.94 ± 0.17^b^
	500	199.80 ± 5.35	5.83 ± 0.11^a^^b^	2.92 ± 0.07^b^

*Values are expressed as means ± S.E.M. of six replicates*.

a *Significant different as compared to normal control, P < 0.05*.

b *Significant different as compared to negative control (10% DMSO + PCM), P < 0.05*.

**Table 6:** Histopathological scoring of PCM-intoxicated rat's liver tissue following pretreatment with AQMC at the doses ranging between 50 and 500 mg/kg.

**Treatment**	**Dose (mg/kg)**	**Steatosis**	**Necrosis**	**Inflammation**	**Haemorrhage**
Normal	−	−	−	−	−
10% DMSO + PCM		−	+++	++	++
NAC + PCM	50	−	+	+	−
AQMC + PCM	50	−	−	++	−
	250	−	−	+	−
	500	−	−	−	−

*The severity of various features of hepatic injury was evaluated based on those following scoring scheme: − normal, + mild effect, ++ moderate effect, +++ severe effect*.

The effect of orally administered AQMC, at 50, 250, and 500 mg/kg, on the level of serum liver enzymes of PILI rats is shown in Table 7. Overall, AQMC caused significant (*p* < 0.05) reduction in the level of serum ALT, AST, and ALP at all doses tested, which was far more effective when compared to the NAC.

**Table 7:** Effect of the orally administered AQMC, at the doses ranging between 50 and 500 mg/kg, on the level of serum liver biomarkers, namely ALT, AST, and ALP, of PCM-intoxicated rats.

**Treatment**	**Dose (mg/kg)**	**ALT (U/L)**	**AST (U/L)**	**ALP (U/L)**
Normal	–	136.05 ± 10.52	124.38 ± 16.14	193.07 ± 41.44
10% DMSO + PCM		1714.71 ± 142.27^a^	2266.07 ± 340.43^a^	330.03 ± 42.35^a^
NAC + PCM	50	884.28 ± 195.40^a^^b^	1569.83 ± 106.49^a^^b^	284.32 ± 5.54
AQMC + PCM	50	507.53 ± 15.96^b^	748.15 ± 201.38^a^^b^	227.29 ± 11.34^b^
	250	297.79 ± 16.93^b^	431.27 ± 16.42^b^	218.79 ± 21.35^b^
	500	63.50 ± 7.24^b^	190.66 ± 33.13^b^	202.04 ± 9.048^b^

*Values are expressed as means ± S.E.M. of six replicates*.

a *Significant different as compared to normal control, P < 0.05*.

b *Significant different as compared to negative control (10% DMSO + PCM), P < 0.05*.

Table 8 shows the effect of orally administered AQMC, at 50, 250, and 500 mg/kg, on the activity of endogenous antioxidant enzymes of PCM intoxicated rats. From the results obtained, PILI leads to decrease in the activity of SOD and CAT when compared to the normal group. Pre-treatment with AQMC was found to significantly (*p* < 0.05) reverse the inhibitory effect of PCM on endogenous SOD and CAT activities leading to increase in the activities of those enzymes at all doses tested. As a comparison, the 50 mg/kg NAC demonstrated an equal effective effect to that of 500 mg/kg AQMC.

**Table 8:** Effect of AQMC, at 50, 250, and 500 mg/kg, on the activities of endogenous antioxidant enzymes, namely SOD and CAT, of PCM- intoxicated rats.

**Treatment**	**Dose (mg/kg)**	**SOD (U/g tissue)**	**CAT (U/g tissue)**
Normal	–	9.36 ± 0.75	122.36 ± 0.74
10% DMSO + PCM		2.42 ± 0.37^a^	68.81 ± 0.56^a^
NAC + PCM	50	10.59 ± 0.47^b^	119.52 ± 1.70^b^
AQMC + PCM	50	4.83 ± 1.71	122.78 ± 4.92^b^
	250	6.61 ± 1.43^b^	115.77 ± 1.01^b^
	500	9.23 ± 0.75^b^	120.63 ± 5.05^b^

*Values are expressed as means ± S.E.M. of six replicates*.

a *Significant different as compared to normal control, P < 0.05*.

b *Significant different as compared to negative control (10% DMSO + PCM), P < 0.05*.

#### UHPLC-UV profile of AQMC

**Figure 3:**
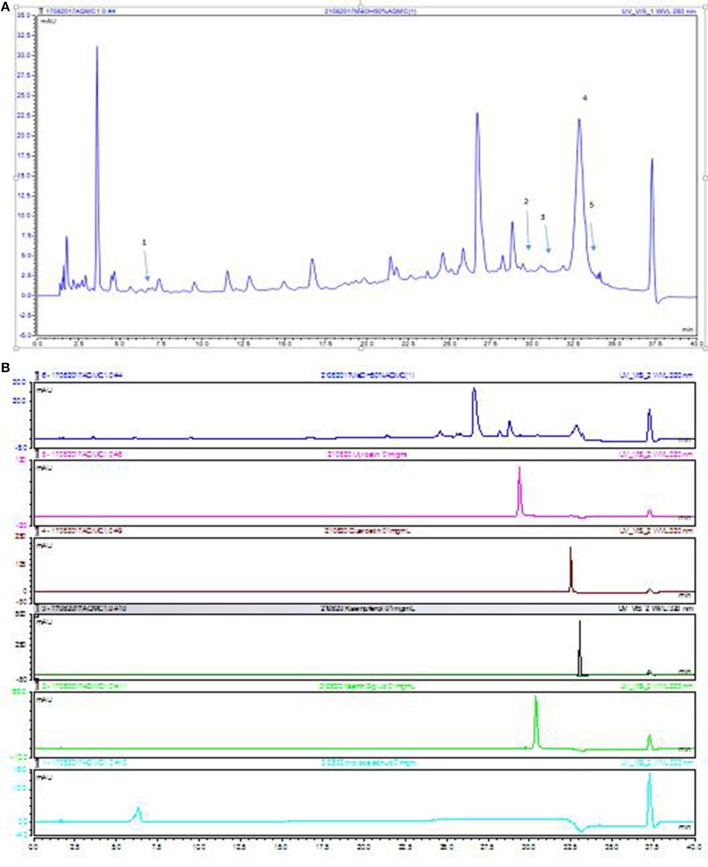
UHPLC-UV of AQMC at 280 nm. **(A)** The numbering of the peaks correspond to those reference standards shown in **(B)** 1. protocatechuic acid, 2.myricetin, 3. kaempferol-3-*O-*glucoside, 4. quercetin and 5. Kaempferol.

The UHPLC-UV profile of AQMC was established at the wavelengths of 280 nm (Figure 3). Five major peaks, labeled as 1, 2, 3, 4 and 5, were identified as protocatechuic acid, myricetin, kaempferol-3-*O-*glucoside, quercetin, and kaempferol.

#### UHPLC-ESI analysis of AQMC

**Figure 4:**
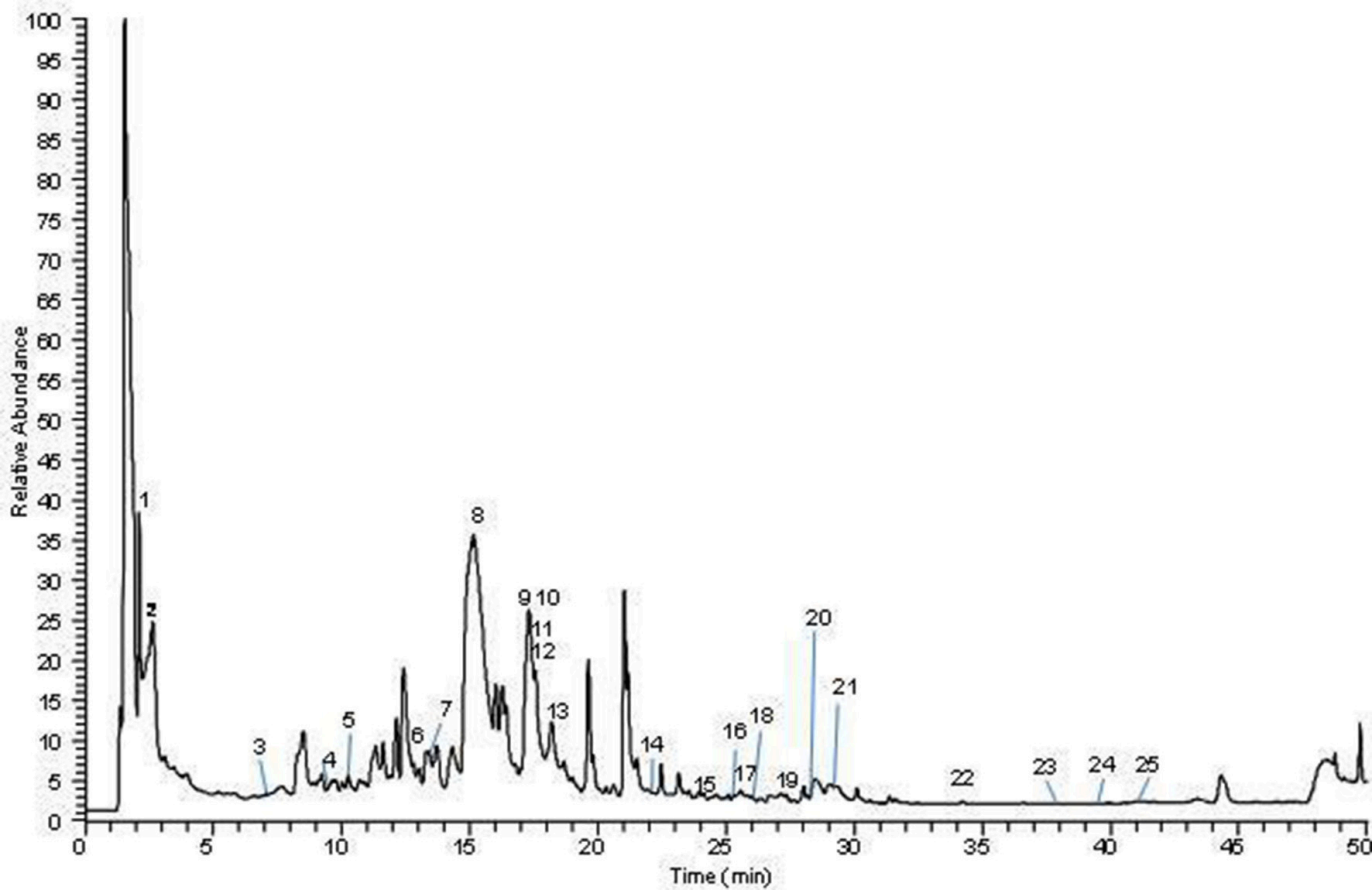
Total ion chromatography (TIC) of the indicated *M.calabura* sample, obtained with the UHPLC-ESI-MS/MS instrument in negative ion mode.

The analysis of AQMC was based on the accurate mass data of the molecular ions, in which ions detected were tentatively identified by their generated molecular formula, through the software Data analysis (Xcalibur) which provided list of possible elemental formulas, together with the use of standard when available and after thorough survey of the literature. The main compounds found in AQMC are organic acids mainly citric and gallic acid and flavonoids glycosides. Iridoids such loganin acid, elenolic acid and 6′-*O*-trans-cinnamoyl-8-epikingisidic acid are also found in the extract which belong to monoterpenoids. The UHPLC-ESI analysis of AQMC revealed the presence of 25 phenolic compounds, which is presented in Figure 4 and Table 9. The table also contains other important information such as the list of peak number, retention time, observed m/z, and the generated molecular formula.

**Table 9:** Phenolic compounds identified in AQMC by UHPLC-ESI-MS/MS.

**Peak**	**t^R^ (min)**	**[M-H]-**	**Error (ppm)**	**Formula**	**Identification**
1	2.63	169.0136	2.723	C_7_H_5_O_5_	Gallic acid
2	2.09	191.0191	2.57	C_6_H_7_O_7_	Citric acid
3	6.85	375.13022	4.390	C_16_H_23_O_10_	Loganin acid
4	9.81	325.0936	5.556	C_15_H_17_O_8_	Coumaryl hexoside
5	10.26	241.07182	4.793	C_11_H_13_O_6_	Elenolic acid
6	13.02	163.0397	4.412	C_9_H_7_O_3_	Protocatechuic acid
7	13.33	317.0306	4.562	C_15_H_9_O_8_	Myricetin
8	15.10	301.0350	2.494	C_15_H_9_O_7_	Quercetin
9	17.27	939.1146	5.125	C_41_H_31_O_26_	Pentagalloyl-hexoside
10	17.33	285.0402	2.58	C_15_H_9_O_6_	Kaempferol
11	17.58	519.5253	5.446	C_25_H_28_O_12_	6′-O-trans-Cinnamoyl-8-epikingisidic acid
12	17.64	599.1055	3.979	C_28_H_23_O_15_	Quercitrin-2″-*O*-gallate
13	18.28	447.0942	4.188	C_21_H_19_O_11_	Kaempferol-3-*O*-galactoside
14	22.01	583.1110	4.679	C_28_H_23_O_14_	Afzelin-O-gallate
15	24.15	269.0819	2.73	C_16_H_13_O_4_	Pinostrobin
16	25.07	593.1318	4.844	C_30_H_25_O_13_	Kaempferol-3-*O*-glucoside i
17	25.56	255.0663	3.586	C_15_H_11_O_4_	Pinocembrin
18	26.02	59.1318	4.945	C_30_H_25_O_13_	Kaempferol-3-*O*-glucoside ii
19	27.35	313.0721	4.617	C_17_H_13_O_6_	Ermanin I
20	27.91	271.0610	2.878	C_15_H_11_O_5_	Pinobaksin
21	29.12	313.0726	4.904	C_17_H_13_O_6_	Ermanin II
22	34.10	269.0818	3.213	C_16_H_13_O_4_	Pinostrobin ii
23	37.97	253.0509	3.138	C_15_H_9_O_4_	Chyrsin i
24	39.62	253.0507	3.852	C_15_H_9_O_4_	Chyrsin ii
25	41.01	299.0564	4.532	C_16_H_11_O_6_	Kaempferide

## Discussion

Due to PCM ability to induce liver toxicity, the PILI assay has been widely accepted and used as an assay to determine potential compound/extract to exert a hepatoprotective activity. Previous study had shown the ability of MEMC to exhibit hepatoprotective activity when assessed using the PILI assay (Mahmood et al., 2014a). Taking into account the potential of MEMC to demonstrate remarkable antioxidant as well as anti-inflammatory activities in addition to the observed hepatoprotective activity, the present study was carried out with an attempt to find a MEMC-based partition with the most effective hepatoprotective activity for future bioactive compound(s) isolation and identification. From the results obtained, PCM successfully induced liver injury indicated by increasing levels of serum liver enzymes (ALT, AST, and ALP) and these findings were further supported by the histopathological analysis of the PCM-intoxicated liver. These can be explicated by the fact that hepatic cells contain a host of enzymes and possess a variety of metabolic activities (Giannini et al., 2005). ALT is found in elevated concentration in cytoplasm while AST is found particularly in mitochondria. The increase in the ALT is usually attended by a rise in the levels of AST. In the case of hepatotoxicity, the transport role of liver cells is disturbed resulting in leakage of those enzymes into the plasma that caused a rise in their serum level. The increased level of ALT and AST in PILI is a sign of cellular leakage and loss of membrane integrity of liver cells (Abou Seif, 2016). Treatment with AQMC or other partitions overturned the increased levels of ALT and AST suggesting the stabilization of plasma membrane and the reversal of PCM-induced hepatic cell damage. Generally, pre-treatment with AQMC at 250 mg/kg was found to effectively attenuate PILI, which was followed sequentially by EAMC and PEMC.

However, the effectiveness of AQMC to attenuate PILI was not in accordance with its TPC value, which was approximately 2-fold less than that of EAMC. Despite this observation, AQMC also exerts equieffective antioxidant activity when compared to EAMC using the DPPH- and SOA-radical scavenging, and ORAC assays. Moreover, AQMC also exerts equal-strength anti-inflammatory activity via LOX pathway when compared to EAMC with the latter also demonstrated remarkable activity via XO pathway. The involvement of LOX in PILI has been studied elsewhere (Pu et al., 2016), wherein 5-LOX was reported to catalyze arachidonic acid to form leukotrienes that lead to oxidative stress and inflammation. The ability of those partitions to inhibit the activity of LOX might partially explain their hepatoprotective potential. With regards to the XO, two contradicting claims were discussed in brief by Du et al. (2016). In the first claim, XO was cited as another potential source of reactive species in the liver following PCM overdose based on two observations, namely: (i) xanthine dehydrogenase was converted to oxidase in the liver after PCM overdose, and (ii) mice pre-treated with XO inhibitor allopurinol attenuated the oxidative stress and liver injury. On the other hand, the second claim argue against XO as a relevant source of reactive species in PILI based on the facts that: (i) the dose of allopurinol needed to protect against PCM intoxication is higher than the dose required to fully inhibit the enzyme, and a lower dose that totally inactivates the enzyme could not lessen the oxidant stress or protect against the toxicity; (ii) the liver protection observes at high doses was actually due to the expression of metallothionein, a product of metabolism of allopurinol, which can react with NAPQI and reactive species, and (iii) neither allopurinol pre-treatment for 1 or 18 h nor oxypurinol (primary metabolite of allopurinol) pre-treatment for 1 h protects against PILI (Du et al., 2016). These claims could be used to explain why AQMC, which inhibit LOX activity more than the XO activity, exerts a more effective hepatoprotective activity in comparison to EAMC, which inhibit both the LOX and XO activities remarkably. It is believed that inhibiting LOX action rather than XO activity will lead to reduce oxidative stress and inflammation resulting in a more effective hepatoprotective activity. Taking into account the link between oxidative and inflammatory processes with PILI (Lawson et al., 2000), it is reasonable to suggest that any compounds/substances which acquire good antioxidant and/or anti-inflammatory activities might also possess significant hepatoprotective potential. Interestingly, AQMC as well as EAMC, which possess both antioxidant and anti-inflammatory activities, might become good candidates to be developed as hepatoprotective agents in the future.

Further investigation also revealed the ability of AQMC to attenuate PILI by modulating the endogenous antioxidant enzymes defense system. PCM was found to cause reduction in the activity of SOD and CAT, and pre-treatment with AQMC was found to restore those enzymes' activity. The importance of endogenous antioxidant enzymes defense system in protecting cellular homeostasis from oxidative disruption by reactive molecules has been discussed by Sanz et al. (2002). The proficient functionality of these defense mechanisms depends on involvement of concerted activity of each individual system. Therefore, SOD, CAT, and other members of this enzymatic defense system must operate co-ordinately in the pro-oxidant cell states. This enzymatic defense system also has to be in unity with the elements accountable for the restore process of oxidatively damaged molecules to preserve the cell integrity (Li et al., 2015). In the present study, AQMC was found to reverse the effect of PCM on the activity of SOD and CAT, thus suggesting the fraction ability to modulate the endogenous enzymatic defense system which may partly contribute to the observed hepatoprotective activity.

Natural products, particularly of plant-based extracts, offer unlimited opportunities for new drug discoveries owing to the unmatched availability of phytochemical diversity (Cosa et al., 2006). These phytochemicals are considered safe and generally are effective alternatives with less adverse effect and can be widely retrieved via various extraction methods. However, the crude plant extract accumulates a combination of various types of phytochemicals with different polarities that require an ideal extraction protocol involving the use of several solvents system to separate the phytochemicals according to their polarity. In the present study, three fractions, namely PEMC, EAMC, and AQMC, were fractionated from the initial crude methanol extract (MEMC), which contain a complex mixture of many plant metabolites as proven using the UHPLC-ESI-MS/MS and GCMS analyses. Initial screening demonstrated the presence of phenolic compounds in all fractions in the sequence of EAMC>PEMC≥AQMC. As highlighted by Zakaria et al. (2014), compounds/extracts with the TPC value of >1,000 mg/100 g GAE can be considered to have high phenolic content and, based on this requirement, all partitions were suggested to have low to moderate amount of phenolic compounds. Moreover, the amount of TPC in AQMC (413 mg/100 g GAE) was also considered low when compared to the amount estimated for MEMC (2751.26 mg/100 g GAE) (Zakaria et al., 2014). The difference in TPC value could be linked to the difference in phytochemical constituents of AQMC and MEMC wherein the former was found to contain only tannins and saponins while the latter has been reported to contain flavonoids in addition to tannins and saponins. Despite the difference in phytoconstituents between the crude extract MEMC in comparison to the partially purified extract AQMC, the flavonoids present in MEMC was qualitatively low. This might explain why flavonoids were not detected in AQMC during the phytochemical screening. However, their presence should not be ignored at all as the HPLC analysis of AQMC did demonstrate the existence of 3 flavonoids peaks; P1, P3, and P5, being the major peak but only at low intensity indicating their fairly low quantity in AQMC. Therefore, it is suggested that the failure of detecting flavonoids in AQMC using the phytochemical screening assay could be attributed to the low concentration of flavonoids presence in AQMC. It is believed that most of the flavonoids were extracted in EAMC, which might explain EAMC's high TPC value in comparison to AQMC and PEMC. The absence or presence of traces of flavonoids in the fraction(s) obtained following the partitioning of methanol crude extract has been reported elsewhere and could be used to explain the present findings (dos Santos et al., 2006).

Analysis of AQMC using HPLC at the wavelength of 280 nm demonstrated the presence of several major peaks, which upon comparison with some pure standard flavonoids, revealed the presence of protocatechuic acid, myricetin, kaempferol-3-*O-*glucoside, quercetin, and kaempferol. Further analysis of AQMC using the UHPLC-ESI leads to the identification of at least 25 peaks, of which some of the major peaks represent compounds that have been reported to demonstrate hepatoprotective activity against PILI such as gallic acid (Rasool et al., 2010), and quercetin (Janbaz et al., 2004). Other than that, compounds like kaempferol (Calderón-Montaño et al., 2011), myricetin (Li and Ding, 2012), pinostrobin (Patel et al., 2016), and pinocembrin (Rasul et al., 2013) have been reported to possess antioxidant and anti-inflammatory activities and are suggested to also take part in enhancing the hepatoprotective activity of AQMC against PILI.

## Conclusion

In conclusion AQMC exerts distinct hepatoprotective activity against PILI via the activation of endogenous antioxidant and LOX-mediated systems. Hepatoprotective activity of AQMC could be attributed partly to the direct action of hepatoprotective-bearing flavonoids, such as gallic acid and quercetin, and to the indirect action of antioxidant- and/or anti-inflammatory-bearing flavonoids, such as kaempferol, myricetin, pinostrobin, and pinocembrin.

## Author contribution

ZZ conceived of the study, participated in its design and, helped to draft and finalized the manuscript. NM, SM, and NN carried out the animal studies, biochemical analysis, statistical analysis and drafted the manuscript. MO performed the HPLC-UV and UHPLC-ESI analyses of the fraction. All authors read and approved the final manuscript.

### Conflict of interest statement

The authors declare that the research was conducted in the absence of any commercial or financial relationships that could be construed as a potential conflict of interest.
